# Supplementary material for: Resveratrol-Loaded Attalea funifera Oil Organogel Nanoparticles: A Potential Nanocarrier against A375 Human Melanoma Cells
Source: Int J Mol Sci. 2023 Jul 28;24(15):12112. doi: 10.3390/ijms241512112 (PMC10419039; doi:10.3390/ijms241512112)

Supplementary Material

Figure S1. *A. funifera* seed oil organogel nanoparticles (**A**) and resveratrol-*A. funifera* seed oil organogel (**B**).

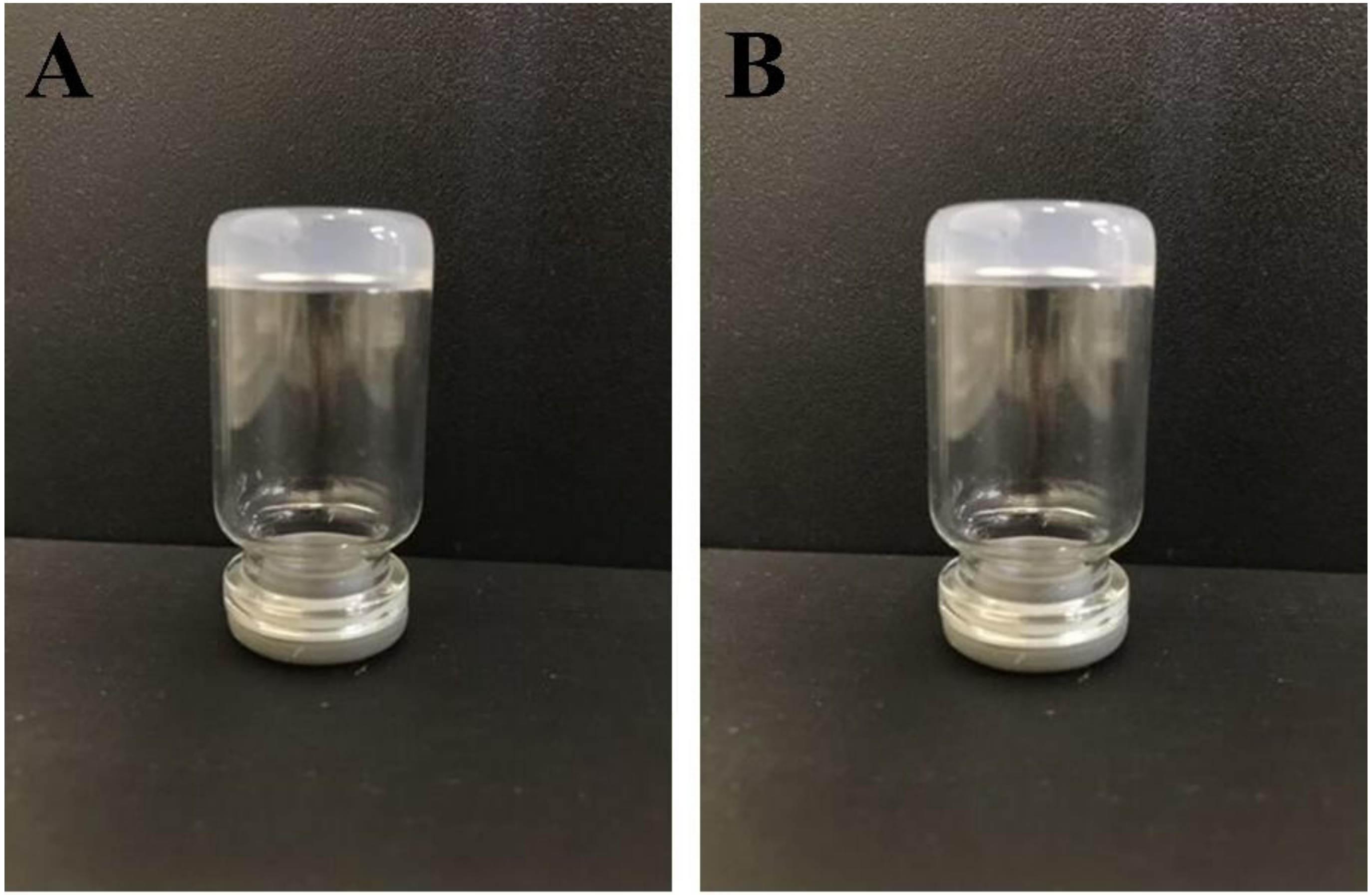

Table S1. Physicochemical characteristics of *Attalea funifera* seed oil.

Parameter	Value*
Acidity index (mgNaOH/g)	0.1770 ± 0.007
Acidity in oleic acid (%)	0.5580 ± 0.060
Peroxide index (mEq/kg)	0.2600 ± 0.037
Refractive index at 20 °C	1.4515 ± < 0.001
Density at 20 °C (g/cm)	0.9202 ± < 0.001

*The values (means ± standard deviation) correspond to averages from three replicates (n = 3).

Figure S2. Scheme of protonation of the resveratrol observed at acid (pH 6) medium (MarvinSketch®, v. 18.11).

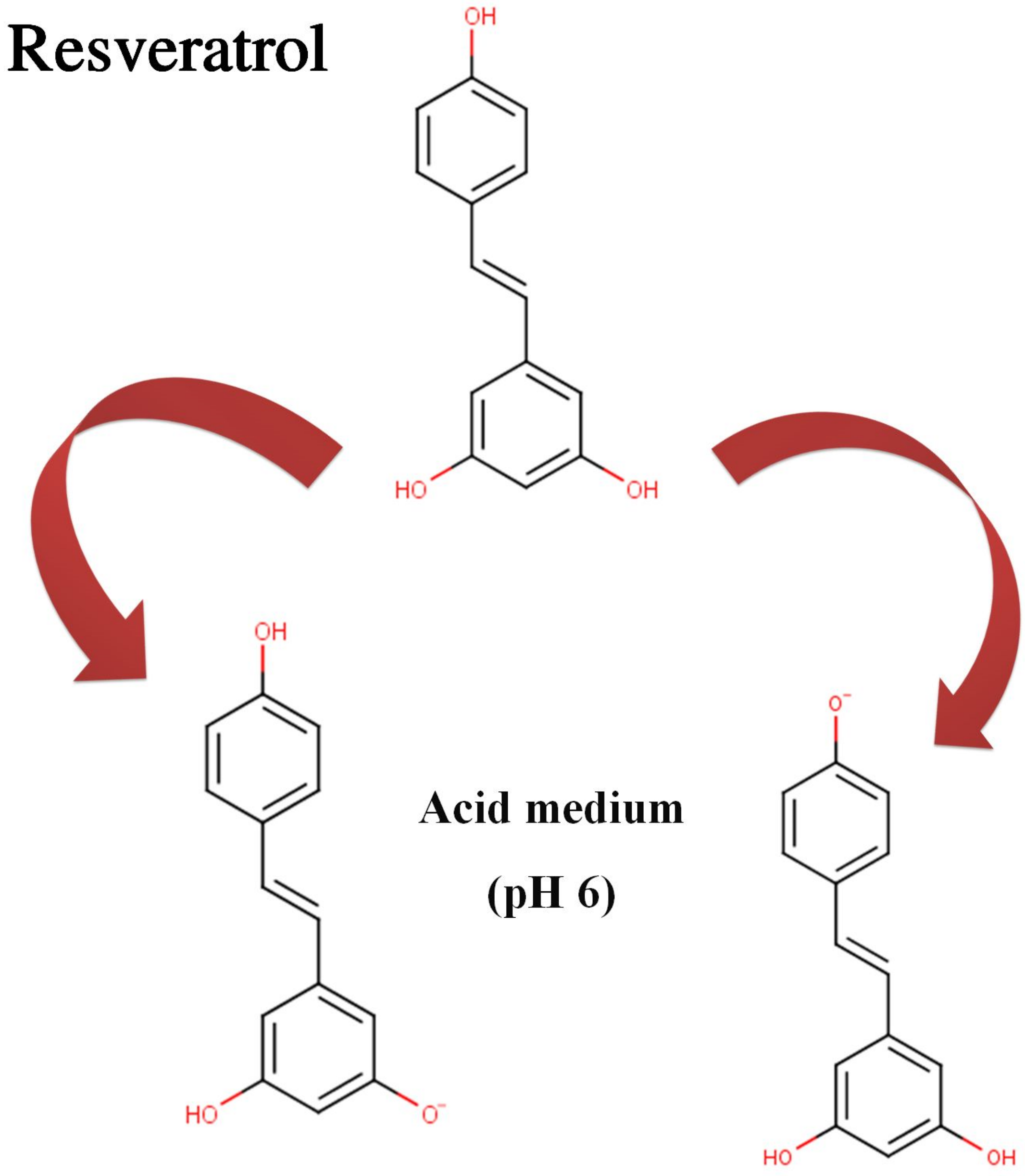

Supplement: Supplementary file 1 [file ijms-24-12112-s001.zip › ijms-2506571-supplementary.pdf]
